# Response to the Commentary from Bevelacqua et al.

**DOI:** 10.1523/ENEURO.0439-19.2019

**Published:** 2020-01-02

**Authors:** Charles L. Limoli, Richard Britten, Janet Baulch, Tomas Borak

**Affiliations:** 1 Department of Radiation Oncology, University of California, Irvine, Irvine, California 92697; 2 Department of Radiation Oncology, Eastern Virginia Medical School, Norfolk, Virginia 23501; 3 Department of Environmental and Radiological Health Sciences, Colorado State University, Fort Collins, Colorado 80523

**Keywords:** commentary, deep space, response, travel

This response clarifies certain misconceptions published in the commentary by Bevelacqua et al. (2019).

We are pleased that our article entitled “New concerns for neurocognitive function during deep space exposures to chronic, low dose rate, neutron radiation” (Acharya et al., 2019) has stimulated interesting discussions and different perspectives on what may or may not be relevant to estimating the risks of CNS dysfunction following exposure to the space radiation environment. Here we provide our response to the commentary from Bevelacqua et al. (2019).

Bevelacqua et al. (2019) stated that there were a few major shortcomings with our approach, and we would like to clarify our stance regarding those statements. The first and perhaps most disconcerting statement was their assertion that we have ignored “…that in a realistic space environment, cells will be exposed to multiple low LET (linear energy transfer) protons before being traversed by intermediate and high-LET HZE (high charge and energy) particles.” The authors of Acharya et al. (2019) have conducted research at heavy ion particle accelerators around the world for more than a decade, and the implication that we might be unaware of the complexities of the radiation fields in space is misguided (Parihar et al., 2015, 2016, 2018; Lee et al., 2017).

During long-term missions into deep space, astronauts will be exposed to a complex radiation field that includes high LET components from high energy, heavy ions (HZE particles) at low dose rates of ∼0.5 mGy/d for long durations. About 20% of the dose is delivered with LET >10 keV/μm.

Particle accelerators are capable of simulating components of the galactic cosmic radiation (GCR) spectrum. The main impediment to performing accelerator-based experiments, which are designed to simulate exposures to high LET radiations during extended missions in space, is that it is not reasonable to irradiate large numbers of rodents continuously over many months at meaningful and relevant doses and dose rates. Fast neutrons from nuclear fission create charged particle nuclei in tissues that have an LET ranging from 10 to 200 keV/μm. We were careful not to imply that the radiation environment in the ^252^Cf facility is an exact replica of conditions within a spacecraft or habitat in deep space. However, the charged particles generated by neutrons are produced uniformly in small animals with a dose-averaged LET that is similar to many of the HZE components of the GCR and about a factor of two higher than space radiation fields resulting from penetrating particles and nuclear fragmentation downstream of shielding.

We disagree with the statement that precision measurements of dose rates and radiation quality for the mixed fields within this facility are “…significantly easier than determining the dose from a spectrum of protons and HZE particles of much greater energy.” Complex dosimetry was required for a large facility capable of exposing 900 mice and 60 rats simultaneously. The radiation field at the exposure location consists of direct neutrons from the source as well as albedo neutrons from the walls and floor. There is also a component of direct photons emitted from the source and scattered photons from the surrounding shielding. Tissue equivalent proportional counters (TEPCs) were used to measure neutrons. A miniature GM (Geiger-Müller) counter and CaF_2_ thermoluminescent dosimeters were used to measure photons. Data from the TEPCs provide direct estimates of the dose rate from neutrons around the room as well as patterns of energy deposition in volumes of tissue similar to the size of a mammalian cell (i.e., lineal energy). These data encompass all the direct and complex environmental modifications to the radiation exposures. Eighty percent of the total dose is from nuclear recoil particles produced by neutron interactions in tissue and 20% is from incident photons. Further details regarding the radiation dosimetry of this facility have been published (Borak et al., 2019).

The authors correctly point out that the animals in the ^252^Cf facility are not subjected to “multiple low LET protons before being traversed by intermediate and high LET particles.” They imply that this sequential process of exposure can lead to the induction of adaptive responses in space. However, it must be noted that in our ^252^Cf facility animals are exposed continuously to low LET photons with intermittent production high LET nuclear recoils.

The assertion that this study should have taken into account the possibility of adaptive responses seems misguided. Adaptive responses have never been convincingly demonstrated *in vivo* and are considered by many radiobiologists to be artifacts of *in vitro* cell culture (Sowa et al., 2010, 2011). To that end, none of the references provided in the commentary documented conclusive evidence regarding the adaptive response *in vivo*, much less in the CNS. Further, the idea that functional CNS end points such as cognition, behavior, and electrophysiology would resemble what has been found using *in vitro* cultures of various cancer cell lines is incongruous. Precisely how *in vitro* studies using transformed cancer cells can shed any light on the functionality of an intact brain is unclear. No agency responsible for radiation protection has incorporated adaptive response into risk assessment models because this effect is not adequately supported in animals or humans.

We are confused with the authors criticism of this work based on the premise that “The absorbed dose does not correspond to a biological detriment.” The suggestion of Bevelacqua et al. (2019) to introduce International Commission on Radiologic Protection stochastic quality factors or radiation weighting factors is meaningless for neurocognitive function. These dose-modifying factors are based on carcinogenesis in humans with most information derived from acute exposures. The objectives of investigations at the ^252^Cf facility are to establish dose, dose rate, and radiation quality effects on biological end points that are beyond the reach of other available experimental protocols. It could be argued that these results will provide valuable information for creating the next generation of dose-modifying factors for radiation risk analysis.

One of the primary driving forces for establishing the neutron facility at Colorado State University was to investigate dose rate effects, since there is a large and reproducible body of literature demonstrating that lowering the dose rate (in cGy/h) provides for the temporal overlap of DNA repair during dose delivery. The end result is that nearly all of the adverse effects of exposure to ionizing radiation are ameliorated at low dose rates simply due to effective DNA repair. It stands to reason then that at space-relevant dose rates similar reductions in adverse effects would be observed, which was not the case in the study by Acharya et al. (2019). It is also important to keep in mind that a critical difference between the study by Acharya et al. (2019) and the references cited by Bevelacqua et al. (2019) is that we did not investigate putative carcinogenic end points in cultured cells. Dose rate effects can be expected to play a relatively minor role in a largely postmitotic organ such as the brain, especially when considering the multifaceted functions that characterize the CNS. At the low doses and dose rates used in Acharya et al. (2019) radiation effects are not likely the result of cell killing, but rather an accumulation of damage to networks of cells that adversely impact neurotransmission.

Regardless of the mechanism, findings from the study by Acharya et al. (2019) demonstrated significant impairments in the brain under the stated irradiation conditions, with no evidence for attenuation by adaptive and/or DNA repair processes over the protracted exposure time. Despite our disagreements with the commentary proffered, we hope these discussions can help the National Aeronautics and Space Administration and other agencies properly evaluate the CNS risks associated with exposure to the deep space radiation environment (Limoli, 2017).

## Synthesis

Reviewing Editor: Mark Baxter, Mount Sinai School of Medicine Decisions are customarily a result of the Reviewing Editor and the peer reviewers coming together and discussing their recommendations until a consensus is reached. When revisions are invited, a fact-based synthesis statement explaining their decision and outlining what is needed to prepare a revision will be listed below. The following reviewer(s) agreed to reveal their identity: Jeffery Chancellor.

Suggested edits:

Lines 12-18 beginning with “A careful review...” These sentences don't contribute to the substantive argument; researchers are capable of publishing numerous papers that are mistaken (not that this is the case here)

Line 28, “ is not feasible to irradiate large numbers of rodents continuously over many months”, is slightly misleading. It is in fact feasible, but not necessarily straight forward or inexpensive.

Lines 64-66 “Adaptive responses ... cell culture” This sentence requires a reference, or if it represents the authors' opinion, should be attributed to the authors instead of “many radiobiologists”

## Author Response

Synthesis of Reviews:

Suggested edits: we have revised all the issues detailed below - please refer to marked copy

Lines 12-18 beginning with “A careful review...“ These sentences don't contribute to the substantive argument; researchers are capable of publishing numerous papers that are mistaken (not that this is the case here)

Line 28, “ is not feasible to irradiate large numbers of rodents continuously over many months”, is slightly misleading. It is in fact feasible, but not necessarily straight forward or inexpensive.

Lines 64-66 “Adaptive responses ... cell culture” This sentence requires a reference, or if it represents the authors' opinion, should be attributed to the authors instead of “many radiobiologists”
